# MIR22HG inhibits breast cancer progression by stabilizing LATS2 tumor suppressor

**DOI:** 10.1038/s41419-021-04105-9

**Published:** 2021-08-26

**Authors:** Xiaochong Deng, Danrong Ye, Kaiyao Hua, Hongming Song, Qifeng Luo, Amik Munankarmy, Diya Liu, Baian Zhou, Wenfang Zheng, Xiqian Zhou, Changle Ji, Xuehui Wang, Yunhe Yu, Lin Fang

**Affiliations:** 1 Department of Breast and Thyroid Surgery, Shanghai Tenth People’s Hospital, Tongji University School of Medicine, Shanghai, 200072 China; 2 Breast Disease Center, The Affiliated Hospital of Qingdao University, Qingdao, 266000 Shandong China

**Keywords:** Breast cancer, Long non-coding RNAs

## Abstract

The long noncoding RNA called MIR22 host gene (MIR22HG) was previously identified as a tumor suppressor in several cancers. However, the biological function of MIR22HG in breast cancer remains unknown. In this study, we aimed to determine the function and molecular mechanism of MIR22HG in breast cancer progression using transcriptomics and biotechnological techniques. Our results showed that *MIR22HG* expression was lower in the cancerous tissues than in the paired adjacent normal breast tissues. Additionally, MIR22HG was found to be mainly located in the cytoplasm and acted as a miR-629-5p sponge. Notably, MIR22HG stabilized the expression of large tumor suppressor 2 (LATS2), which promoted the LATS2-dependent phosphorylation of YAP1 and suppressed the expression of its downstream target oncogenes, thereby inhibiting the proliferation and migration of breast cancer cells. Therefore, our findings reveal the MIR22HG-dependent inhibition of breast cancer cell proliferation and migration via the miR-629-5p/LATS2 pathway, providing new insights and identifying novel therapeutic targets for breast cancer treatment.

## Introduction

As the most commonly diagnosed cancer worldwide, female breast cancer had an estimated incidence rate of 11.7% in 2020 and was known to greatly influence women’s health [1]. Previous studies have successfully identified several biomarkers and therapeutic targets for breast cancer treatment. In addition, increasing evidences show that long noncoding RNAs (lncRNAs) play important roles in breast cancer progression [2].

LncRNAs are defined as noncoding RNA molecules with more than 200 nucleotides [3]. Several lncRNAs, such as MALAT1 [4], RAB11B-AS1 [5], TROJAN [6], and HOST2 [7], have been found to facilitate the proliferation and metastasis of breast cancer cells. MALAT1 binds to the transcription factor TEAD and inactivates the transcriptional activity of the YAP/TEAD complex, subsequently inhibiting the metastatic ability of breast cancer cells [4]. The HIF2-induced RAB11B-AS1 recruits RNA polymerase II to upregulate *VEGFA* and *ANGPTL4* expression, consequently promoting tumor angiogenesis and distant metastasis of breast cancer [5]. In triple-negative breast cancer, TROJAN interacts with metastasis-repressing factor ZMYND8, subsequently increasing ZMYND8 degradation via the ubiquitin-proteasome pathway by repelling ZNF592, and thus enhancing cancer progression [6]. In our previous research, HOST2 was revealed to act as a competitive endogenous RNA via decoying of let-7b, thereby promoting STAT3-mediated cell proliferation and migration in triple-negative breast cancer [7].

Notably, the lncRNA called MIR22 host gene (MIR22HG) has also been observed in several tumors. The tumor suppression function of MIR22HG has been described in hepatocellular carcinoma [8], colorectal cancer [9], and glioblastoma [10]. However, the function of MIR22HG in breast cancer remains unknown. Hence, this study aimed to determine the function and molecular mechanism of MIR22HG in breast cancer progression using transcriptomics and biotechnological techniques.

## Results

### *MIR22HG* expression is downregulated in breast cancer tissues

**Fig. 1:**
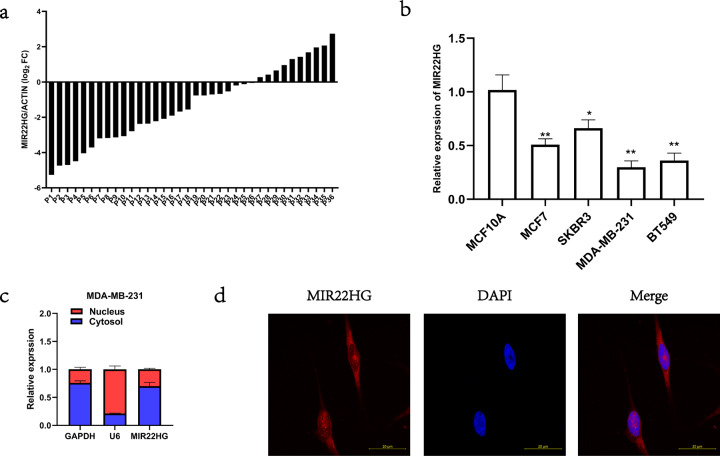
Expression and subcellular distribution of *MIR22HG*. **a***MIR22HG* expression in 36 pairs of cancerous and adjacent normal breast tissues detected by real-time PCR, with β-actin as internal control. **b** *MIR22HG* expression in normal breast epithelial cell line MCF10A and breast cancer cell lines MCF7, MDA-MB-231, BT549, and SKBR3 detected by real-time PCR, with β-actin as internal control. **c** *MIR22HG* expression in subcellular fractions of MDA-MB-231 cells detected by real-time PCR, with *U6* and *GAPDH* as nuclear and cytoplasmic markers, respectively. **d** Fluorescence in situ hybridization analysis revealed that MIR22HG (red) was mainly located in the cytoplasm. DAPI-stained nuclei are in blue (in 63*oil lens). *P < 0.05, **P < 0.01. All experiments were repeated thrice.

To investigate *MIR22HG* expression in breast cancer tissues, we collected 36 pairs of cancerous tissues and adjacent normal breast tissues for real-time PCR analysis. Our results showed that in most paired tissues (26/36), *MIR22HG* expression was lower in cancerous tissues than in normal tissues (Fig. 1a). Similarly, the MIR22HG expression was lower in the studied breast cancer cell lines, namely MCF7, MDA-MB-231, BT549, and SKBR3, than in the normal breast epithelial cell line MCF10A (Fig. 1b). In addition, to determine the subcellular localization of *MIR22HG* in breast cancer cells, we separated the nuclear and cytoplasmic RNA of MDA-MB-231 cells. The real-time PCR results indicated that *MIR22HG* was mainly localized in the cytoplasm (Fig. 1c). Further analysis via fluorescence in situ hybridization also confirmed that *MIR22HG* was located in the cytoplasm (Fig. 1d).

### Loss-of-function of *MIR22HG* promotes breast cancer cell proliferation and migration in vitro

**Fig. 2:**
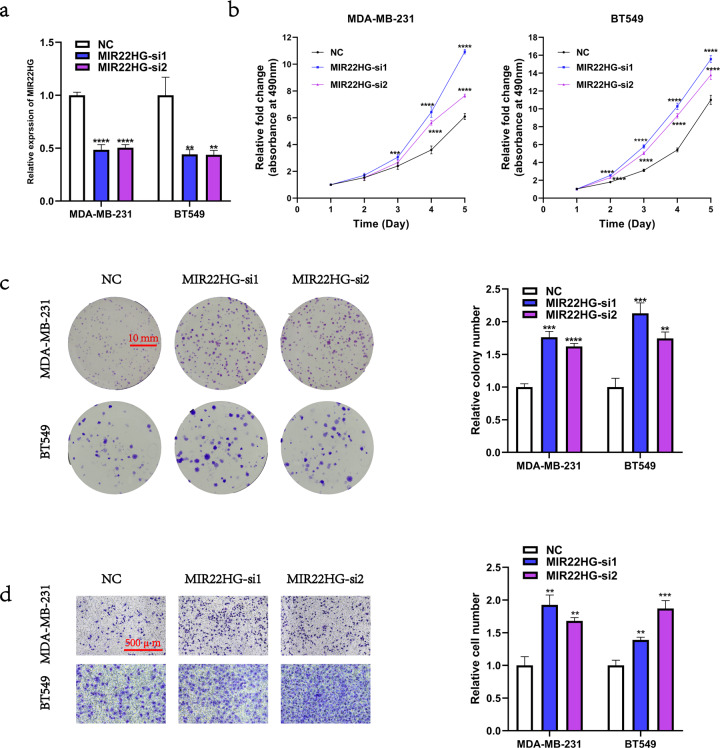
Loss-of-function of *MIR22HG* promotes the proliferation and migration of breast cancer cells. **a** Knockdown efficiency was detected by real-time PCR after transfection of MDA-MB-231 and BT549 cells with siRNAs targeting *MIR22HG* (*MIR22HG*-si1 and *MIR22HG*-si2) or negative control (NC) for 48 h. **b**, **c** MTT assay (5-day period) and colony formation assay (18-day period) were used to determine the viability of MDA-MB-231 and BT549 cells after *MIR22HG* knockdown. **d** Transwell assay (20-h period, 3 × 10^4^ cells per well) was performed to evaluate the migration ability of MDA-MB-231 and BT549 cells after *MIR22HG* knockdown (100 × magnification). **P < 0.01, ***P < 0.001, ****P < 0.0001. All experiments were repeated thrice.

To determine the biological function of MIR22HG in breast cancer cells, we transfected MDA-MB-231 and BT549 cells with *MIR22HG*-siRNAs. Considering the off-target effect of siRNAs, the *MIR22HG*-si1 and *MIR22HG*-si2 were used. The knockdown efficiency of *MIR22HG*-si1 and *MIR22HG*-si2 were detected by real-time PCR (Fig. 2a). After *MIR22HG* knockdown, the changes in the cell proliferation ability were detected by MTT assays and colony formation assays, while the changes in the cell migration ability were analyzed by Transwell assays. Notably, our results showed that loss-of-function of *MIR22HG* enhanced the proliferation (Fig. 2b, c) and migration (Fig. 2d) abilities of breast cancer cells.

### Gain-of-function of *MIR22HG* inhibits breast cancer cell proliferation and migration in vitro and in vivo

**Fig. 3:**
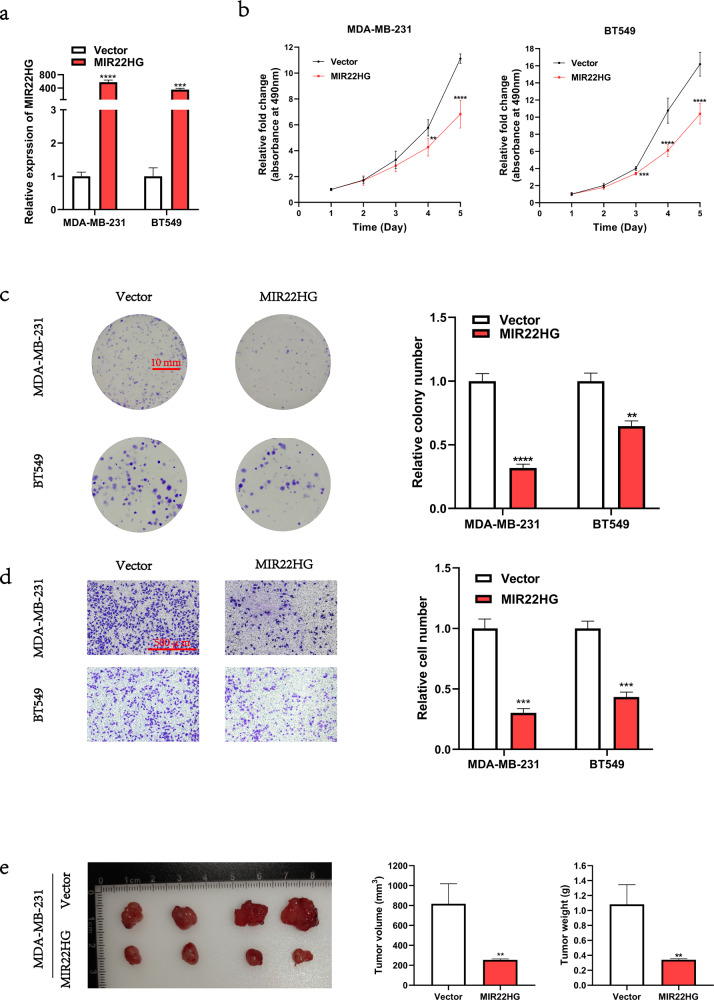
Gain-of-function of *MIR22HG* inhibits the proliferation and migration of breast cancer cells. **a** Overexpression efficiency of *MIR22HG* in MDA-MB-231 and BT549 cells after transfection with *MIR22HG* pcDNA3.1 plasmid for 48 h. **b**, **c** MTT assay (5-day period) and colony formation assay (18-day period) were performed to determine the viability of MDA-MB-231 and BT549 cells after transfection. **d** Transwell assay (20-h period, 3 × 10^4^ cells per well) was used to evaluate the migration ability of MDA-MB-231 and BT549 cells after *MIR22HG* overexpression (100× magnification). **e** A subcutaneous xenograft model was utilized to explore the biological function of *MIR22HG* in vivo. The MDA-MB-231 cells that stably expressed *MIR22HG* were subcutaneously injected into 6-week-old BALB/c nu/nu female mice. After 30 days, the tumors were collected, and the tumor volume and weight were measured. **P < 0.01, ***P < 0.001, ****P < 0.0001. All experiments were repeated thrice.

In addition to *MIR22HG* knockdown, we transfected MDA-MB-231 and BT549 cells with *MIR22HG* plasmids to achieve overexpression. The overexpression efficiency was detected by real-time PCR (Fig. 3a). Results showed that *MIR22HG* overexpression suppressed the proliferation (Fig. 3b, c) and migration (Fig. 3d) of breast cancer cells. Furthermore, we constructed MDA-MB-231 cells that stably expressed *MIR22HG* and a subcutaneous xenograft model to explore the biological function of MIR22HG in vivo. Compared to wild-type MDA-MB-231 cells, the tumorigenicity of MDA-MB-231 cells with stable *MIR22HG* expression was suppressed (Fig. 3e). Collectively, our results suggest that gain-of-function of *MIR22HG* inhibited the proliferation of breast cancer cells both in vitro and in vivo.

### MIR22HG functions as an miR-629-5p sponge in breast cancer cells

**Fig. 4:**
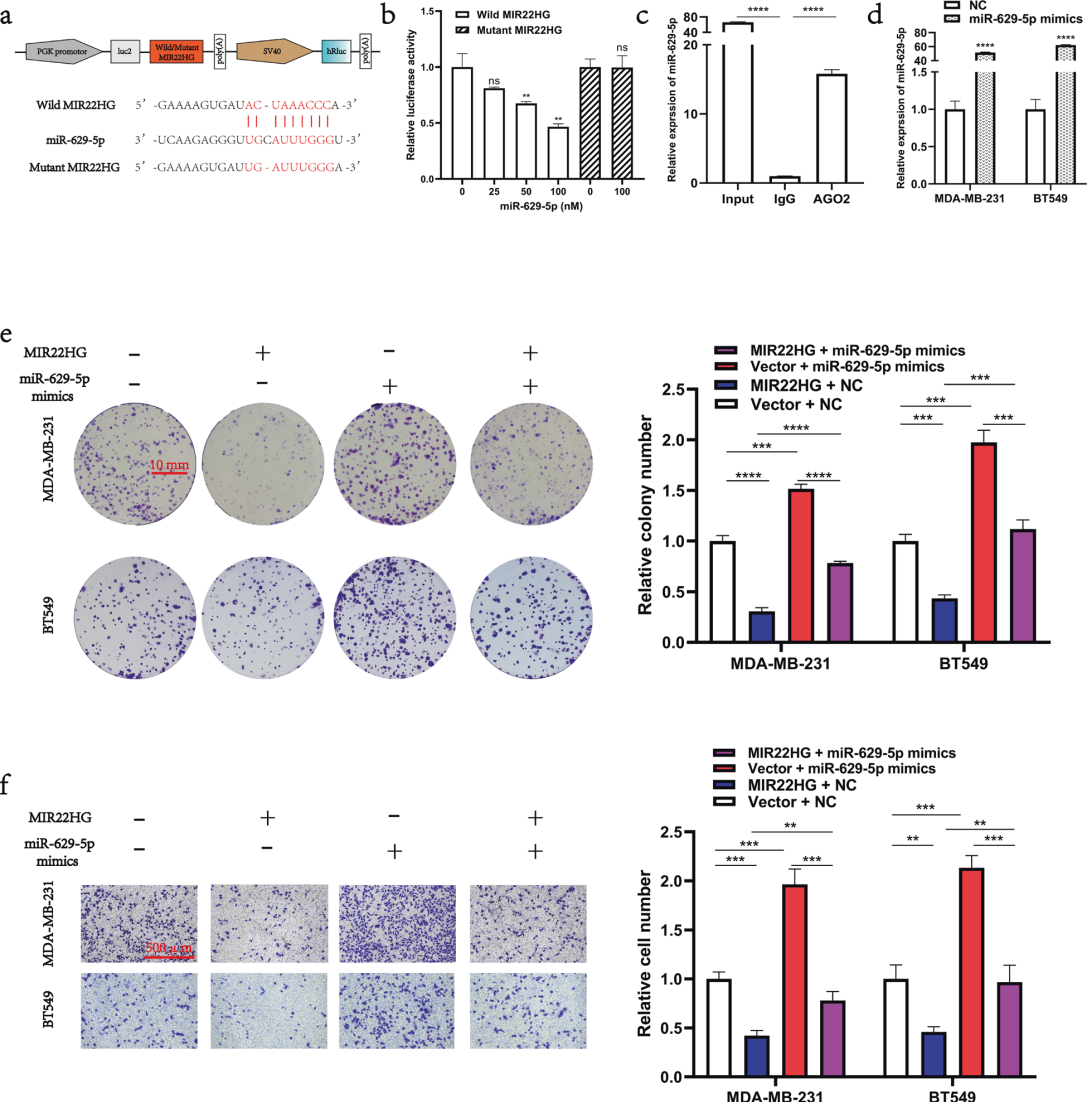
MIR22HG functions as an miR-629-5p sponge in breast cancer cells. **a** Schematic diagram of the interaction between miR-629-5p and wild-type/mutant MIR22HG. **b** Dual-luciferase assays in HEK293T cells co-transfected with wild-type/mutant MIR22HG and miR-629-5p mimics (25, 50, and 100 nM) or NC. At 48 h after transfection, the 293 T cells were collected for the detection of firefly luciferase activity, with Renilla luciferase activity as internal control. **c** AGO2 immunoprecipitation assay was performed using HEK293T cells to detect the expression of miR-629-5p associated with AGO2. The AGO2-specific antibody was applied to precipitate the AGO2-RNA complex after *MIR22HG* overexpression in 293T cells. Total RNA was extracted, and real-time PCR was performed to determine the expression of miR-629-5p associated with AGO2, using IgG-specific antibody as negative control. **d** Overexpression efficiency of miR-629-5p was analyzed by real-time PCR after MDA-MB-231 and BT549 cells were transfected with miR-629-5p mimics or NC. **e** The effects of MIR22HG and miR-629-5p co-transfection on the proliferation of MDA-MB-231 and BT549 cells were evaluated by colony formation assay (18-day period). **f** The effects of MIR22HG and miR-629-5p co-transfection on the migration of MDA-MB-231 and BT549 cells were evaluated by Transwell assay (20-h period, 3 × 10^4^ cells per well, 100× magnification). **P < 0.01, ***P < 0.001, ****P < 0.0001. All experiments were repeated thrice.

**Fig. 5:**
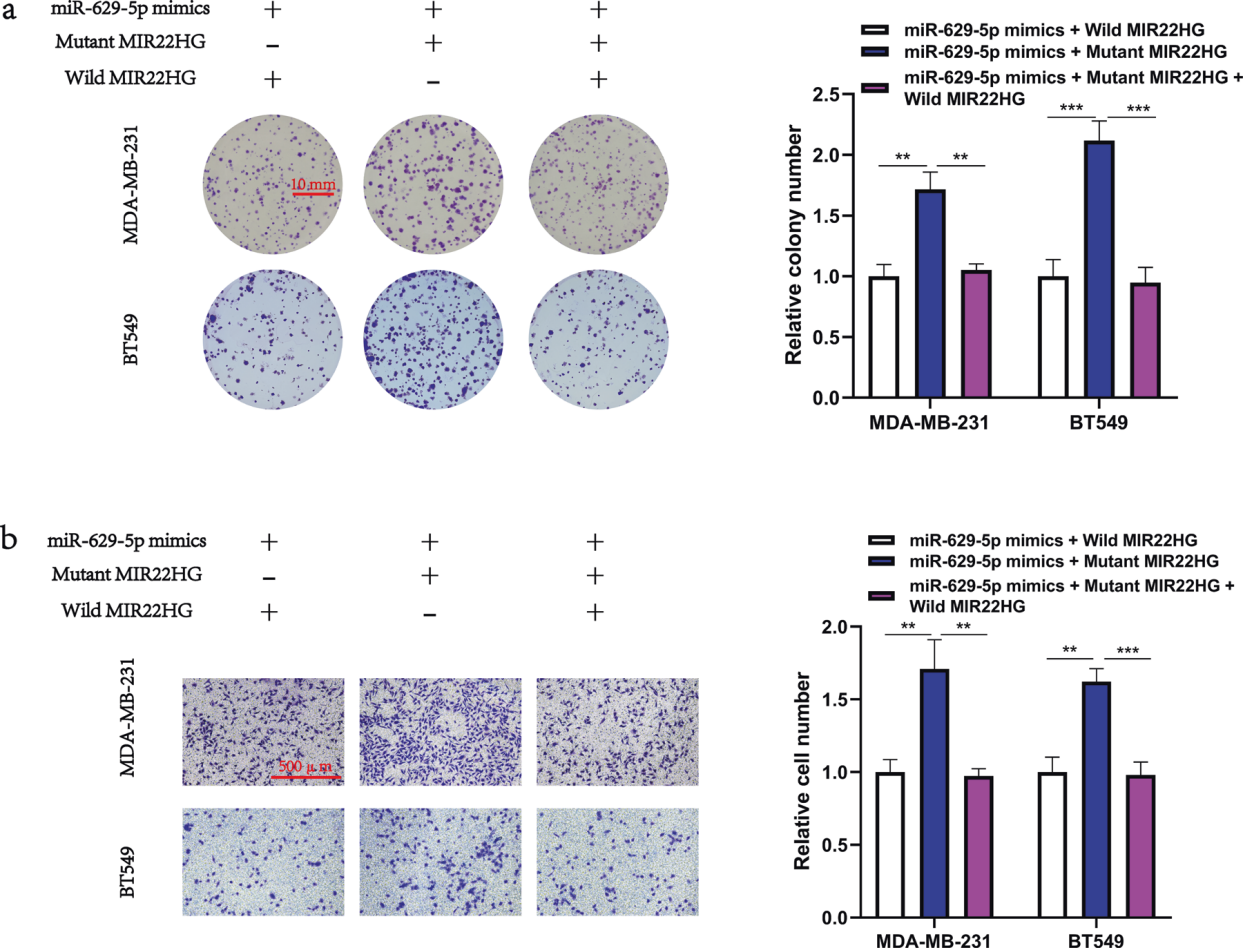
Only wild-type MIR22HG functions as an miR-629-5p sponge in breast cancer cells. **a** Breast cancer cells co-transfected with miR-629-5p and wild-type/mutant MIR22HG. Colony formation assay was used to assess the proliferation ability of MDA-MB-231 and BT549 cells. **b** Transwell assay was performed to assess the migration ability of MDA-MB-231 and BT549 cells. **P < 0.01, ***P < 0.001. All experiments were repeated thrice.

Since MIR22HG was mainly located in the cytoplasm, we hypothesized that MIR22HG may function as a miRNA sponge in breast cancer cells. Hence, we searched the starBase database [11] (http://starbase.sysu.edu.cn/) and discovered that miR-629-5p was a potential target of MIR22HG. To validate the relationship between MIR22HG and miR-629-5p, we constructed the pmirGLO–*MIR22HG*–wild-type and pmirGLO–*MIR22HG*–mutant vectors for subsequent luciferase reporter assay (Fig. 4a). Our results indicated that miR-629-5p reduced the luciferase activity of the pmirGLO–*MIR22HG*–wild-type vector and failed to bind with the mutant MIR22HG (Fig. 4b). In contrast, the immunoprecipitation assay demonstrated that AGO2 was able to pull down both MIR22HG and miR-629-5p (Fig. 4c). Taken together, these results suggest that MIR22HG may act as a competing endogenous RNA molecule to suppress miR-629-5p. We then investigated the function of miR-629-5p in breast cancer cells. The MDA-MB-231 and BT549 cells were transfected with miR-629-5p mimics, and the overexpression efficiency of miR-629-5p mimics was detected by real-time PCR (Fig. 4d). We discovered that miR-629-5p overexpression promoted the proliferation and migration of both MDA-MB-231 and BT549 cells. Notably, results of the rescue experiment suggest that MIR22HG impaired the function of miR-629-5p in breast cancer cells (Fig. 4e, f). Furthermore, the colony formation assay and Transwell assay results of breast cancer cells co-transfected with miR-629-5p and wild-type/mutant MIR22HG revealed that only the wild-type MIR22HG impaired the function of miR-629-5p in breast cancer cells (Fig. 5a, b). Collectively, our results suggest that MIR22HG act as a miRNA sponge that can inhibit function of miR-629-5p in breast cancer cells.

### MIR22HG regulates *LATS2* expression via inhibition of miR-629-5p

**Fig. 6:**
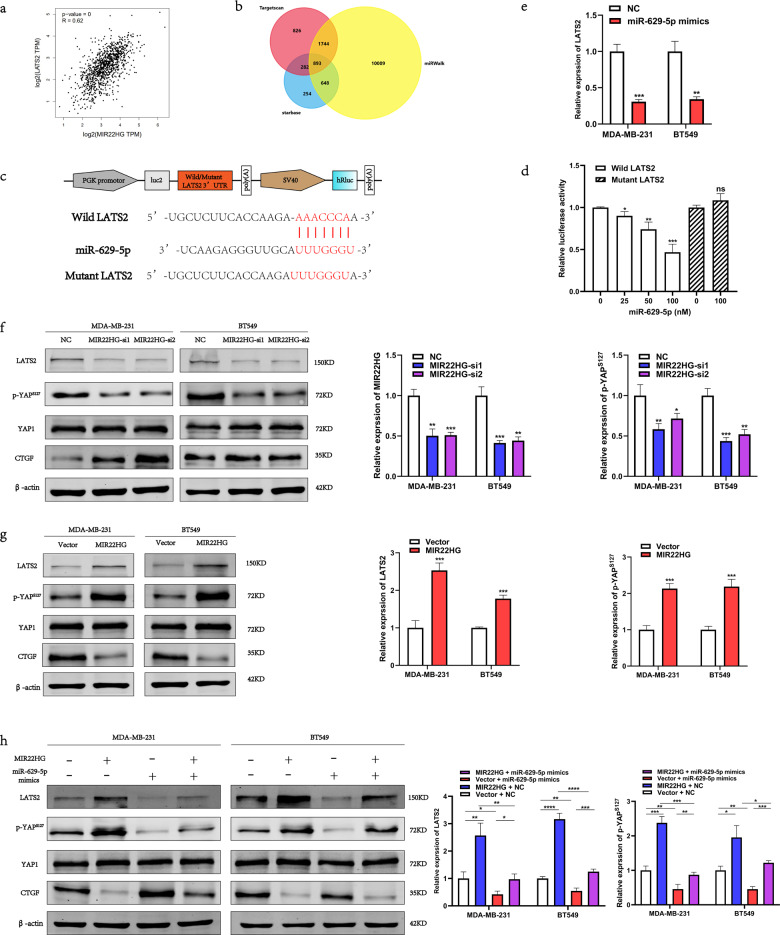
MIR22HG regulates *LATS2* expression via inhibition of miR-629-5p activity. **a** The expression correlation between *MIR22HG* and *LATS2* based on the Gene Expression Profiling Interactive Analysis 2 (GEPIA2) database. **b** Venn diagram showing the overlapping target genes of miR-629-5p predicted using the miRWalk, starBase, and TargetScan databases. **c** Schematic diagram of the interaction between miR-629-5p and wild-type/mutant *LATS2* 3′-UTR. **d** Dual-luciferase assays of HEK293T cells co-transfected with wild-type/mutant *LATS2* 3′-UTR and miR-629-5p mimics (25, 50, and 100 nM) or NC. At 48 h after transfection, the 293 T cells were collected for the detection of firefly luciferase activity, with Renilla luciferase activity as internal control. **e** Real-time PCR was performed to detect the changes in *LATS2* expression in MDA-MB-231 and BT549 after transfection with miR-629-5p mimics for 48 h. **f**, **g** Expression of Hippo-YAP1 signals, namely LATS2, YAP1, phosphorylated YAP1 (S127), and CTGF, was assessed by Western blotting after *MIR22HG* knockdown or overexpression. **h** Western blot analysis revealed that miR-629-5p inhibited the expression of *LATS2*, while *MIR22HG* was found to slightly counteract the miR-629-5p-induced reduction in LATS2 expression. *P < 0.05, **P < 0.01, ***P < 0.001, ****P < 0.0001. All experiments were repeated thrice.

**Fig. 7:**
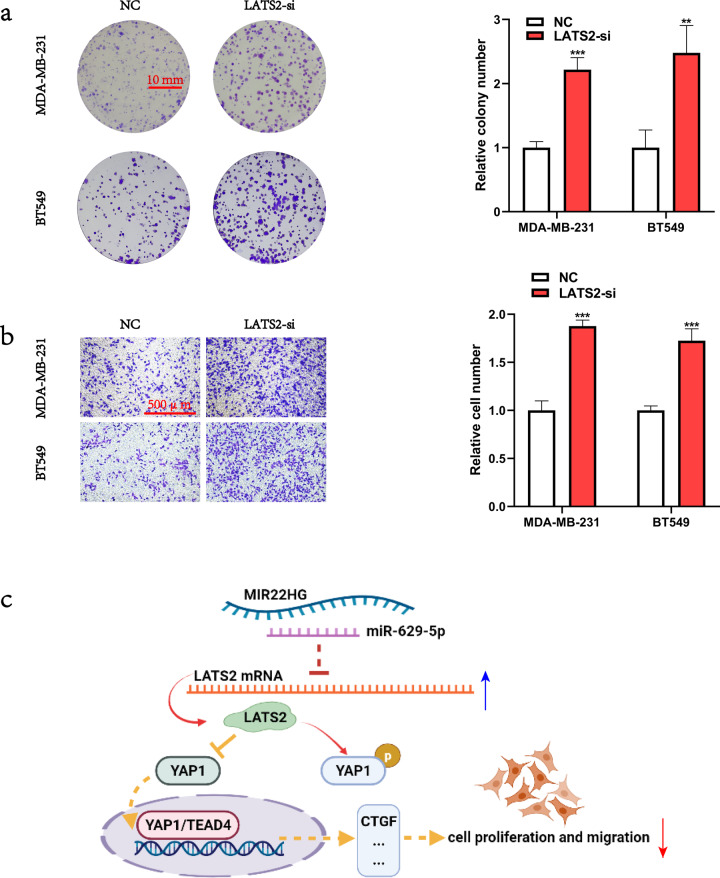
*LATS2* knockdown promoted the proliferation and migration of breast cancer cells. **a** Colony formation assay was used to determine the viability of MDA-MB-231 and BT549 cells after *LATS2* knockdown. **b** Transwell assay was performed to evaluate the migration ability of MDA-MB-231 and BT549 cells after *LATS2* knockdown (100× magnification). **c** A scheme of the proposed molecular mechanisms. MIR22HG stabilizes LATS2 by counteracting the miR-629-5p-induced degradation of *LATS2* mRNA, consequently resulting in the promotion of LATS2-dependent YAP1 phosphorylation and YAP1 inactivation. ***P < 0.001. All experiments were repeated thrice.

To identify the downstream targets of MIR22HG and miR-629-5p, we analyzed the breast cancer data from The Cancer Genome Atlas (TCGA) database and found that *LATS2* expression was positively correlated with *MIR22HG* expression [12] (Fig. 6a). Additionally, utilization of the miRWalk [13] (http://mirwalk.umm.uni-heidelberg.de/), starBase [11] (http://starbase.sysu.edu.cn/), and Targetscan [14] (http://www.targetscan.org/) databases revealed LATS2 as a potential downstream target of miR-629-5p (Fig. 6b). To confirm the relationship between miR-629-5p and LATS2, we constructed the pmirGLO–*LATS2* 3′-UTR–wild-type and pmirGLO–*LATS2* 3′-UTR–mutant vectors (Fig. 6c) and then performed luciferase reporter assay. Our results demonstrated that miR-629-5p can bind with *LATS2* 3′-UTR (Fig. 6d), which promoted the degradation of *LATS2* mRNA (Fig. 6e). We also examined the changes in the expression of LATS2 protein via Western blotting and found that miR-629-5p inhibited LATS2 expression, while MIR22HG slightly counteracted the miR-629-5p-induced decrease in LATS2 expression. As a key member of the Hippo-YAP1 signaling pathway, LATS2 can function as an upstream kinase of the transcriptional cofactor YAP1, which facilitates YAP1 phosphorylation and inactivates the transcriptional activity of the YAP1/TEAD4 complex. Hence, we analyzed the changes in the expression of Hippo pathway signals—CTGF, phosphorylated YAP1, and total YAP1—following *MIR22HG* knockdown or overexpression. The results showed that *MIR22HG* promoted YAP1 phosphorylation and slightly counteracted the miR-629-5p-induced decrease in YAP1 phosphorylation, while no changes were observed in total YAP1 expression (Fig. 6f–h). Finally, investigation of the biological function of LATS2 in breast cancer revealed that the siRNA-induced knockdown of *LATS2* enhanced the proliferation and migration of breast cancer cells (Fig. 7a, b), indicating that LATS2 was also a tumor suppressor in breast cancer. Taken together, our results suggest that MIR22HG acts as a miR-629-5p sponge that can inhibit the proliferation and migration of breast cancer cells and stabilize LATS2 expression.

## Discussion

To determine the function of MIR22HG in breast cancer, we investigated 36 pairs of cancerous tissues and adjacent normal breast tissues via real-time PCR. The results showed that *MIR22HG* expression was lower in cancerous tissues than in normal tissues. The subcellular distribution of lncRNAs determines their biological function [15]. For example, BCRT1, which is mainly located in the cytoplasm, can competitively bind with miR-1303 [16]. In contrast, BX111, which is primarily located in the nucleus, binds with transcription factor YB1 to regulate the expression of its downstream target genes [17]. In the present study, we discovered that MIR22HG was mainly distributed in the cytoplasm, indicating that MIR22HG may act as a competitive endogenous RNA molecule. Subsequent bioinformatic analysis revealed that MIR22HG can bind with miR-629-5p. Furthermore, the luciferase reporter assay and AGO2 immunoprecipitation assay results confirmed that MIR22HG can act as a miRNA sponge and directly bind with miR-629-5p. Functional analysis via siRNA-induced knockdown of *MIR22HG* resulted in the enhanced proliferation and metastasis of breast cancer cells, while *MIR22HG* overexpression inhibited cancer cell proliferation and metastasis. In the Hippo-YAP1 signaling pathway, LATS2 plays a critical role in the phosphorylation and inactivation of the transcription cofactor YAP1/TAZ. The phosphorylated YAP1 is degraded in the cytoplasm by the ubiquitin-proteasome system, consequently inhibiting the expression of YAP1 target genes that are needed for cell proliferation and migration [18]. Taking these into consideration, our results suggest that LATS2 also functions as a tumor suppressor in breast cancer and that miR-629-5p can promote the proliferation and migration of breast cancer cells by targeting *LATS2* mRNA. Additionally, MIR22HG can stabilize *LATS2* expression by counteracting the miR-629-5p-induced degradation of *LATS2* mRNA (Fig. 7c). Therefore, our research reveals the biological function and molecular mechanism of MIR22HG in breast cancer cells and provides novel insights and therapeutic targets for future studies on breast cancer treatment.

## Materials and methods

### Tissue samples

The 36 pairs of cancerous tissues and adjacent normal breast tissues used in this study were collected from the Department of Breast and Thyroid Surgery, Shanghai Tenth People’s Hospital (Shanghai, China). The study was conducted in accordance with the Declaration of Helsinki (as revised in 2013) and approved by the ethics board of Shanghai Tenth People’s Hospital (No. 2020-KN174-01). All study participants were contacted by telephone to obtain verbal informed consent. After collection via surgery, the cancerous and normal tissues were cryopreserved in liquid nitrogen for temporary storage.

### Cell culture

The human embryonic kidney cell line HEK293T, human breast cancer cell lines MCF7, MDA-MB-231, BT549, and SKBR3, and human normal breast epithelial cell line MCF10A were purchased from the Chinese Academy of Science, Shanghai (China). The MCF7, MDA-MB-231, and 293 T cells were cultured in DMEM medium with high glucose (Gibco, Thermo Fisher Scientific, Waltham, MA, USA), while the BT549 and MCF10A cells were cultured in RPMI-1640 medium (Gibco, USA) and mammary epithelial cell medium (Procell, Wuhan, China), respectively. The growth media were added with 10% fetal bovine serum (Gibco, USA) and 100 U/mL penicillin and 100 μg/mL streptomycin (Servicebio, Wuhan, China). A 0.25% trypsin-EDTA solution (Servicebio, China) was also applied for cell passage cultivation. All cell lines were cultivated in a cell incubator at 37 °C and 5% CO_2_.

### Cell transfection

The *MIR22HG*-siRNAs (*MIR22HG*-si1, Sense: 5′-GAGUAGAAGGCUCAAACAACC-3′ and Antisense: 5′-UUGUUUGAGCCUUCUACUCCU-3′; *MIR22HG*-si2, Sense: 5′-CAGGAAAUUCAUAAAAGAAAU-3′ and Antisense: 5′-UUCUUUUAUGAAUUUCCUGUG-3′), pcDNA3.1-*MIR22HG* plasmid, miR-629-5p mimics, large tumor suppressor 2 (*LATS2*)-siRNA (*LATS2*-si, Sense: 5′-GAGUGAUAAUCUUCAAAAUGA-3′ and Antisense: 5′-AUUUUGAAGAUUAUCACUCUC-3′), negative control (NC) siRNA (Sense: 5′-UUCUCCGAACGUGUCACGUTT-3′ and Antisense: 5′-ACGUGACACGUUCGGAGAATT-3′), and pcDNA3.1-vector were transfected into MDA-MB-231 and BT549 cells using Lipofectamine^®^ 3000 reagent (Invitrogen, Thermo Fisher Scientific, Waltham, MA, USA). All sequences were synthesized by Generay Biotechnology (Shanghai, China).

### RNA extraction and cDNA synthesis

Total RNA was extracted from MCF10A, MDA-MB-231, BT549, SKBR3, and 293 T cells and from cancerous tissues and paired adjacent normal breast tissues using TRIzol^®^ Reagent (Invitrogen, USA). For first strand cDNA synthesis, 1000 ng total RNA was reverse-transcribed using Hifair^®^ III 1st Strand cDNA Synthesis SuperMix for qPCR (Yeasen, Shanghai, China) and miRNA 1st Strand cDNA Synthesis Kit (Vazyme, Nanjing, China), following the manufacturers’ instructions.

### Subcellular fractionation

The nuclear and cytoplasmic separation in cells were performed using PARIS Kit (Ambion, Life Technologies, Grand Island, NY, USA), following the manufacturer’s protocols.

### Fluorescence in situ hybridization

The subcellular localization of *MIR22HG* in MDA-MB-231 cells was analyzed using Fluorescent In Situ Hybridization Kit (RiboBio, Guangzhou, China), according to the manufacturer’s instructions. The Cy3-labeled MIR22HG probes were designed and synthesized by RiboBio (China). Representative images were obtained using a confocal microscope (Carl Zeiss Jena, Germany).

### Real-time PCR

Real-time PCR was performed using Hieff UNICON^®^ qPCR SYBR Green MasterMix (Yeasen, China), with β-actin as internal control for normalizing *MIR22HG* and *LATS2* expression and *U6* as internal control for normalizing miR-629-5p expression. All primers were synthesized by Generay Biotechnology (China). The primers used for qPCR include the following: *MIR22HG* (Forward: 5′-CCATACATTGCGTGTGGGAG-3′, Reverse: 5′-TTCGTAGGTCAAATGACATGGAG-3′); β-actin (Forward: 5′-CATGTACGTTGCTATCCAGGC-3′, Reverse: 5′-CTCCTTAATGTCACGCACGAT-3′); Hsa_miR-629-5p (Forward: 5′-GCGTGGGTTTACGTTGGG-3′, Reverse: 5′-AGTGCAGGGTCCGAGGTATT-3′, RT: 5′-GTCGTATCCAGTGCAGGGTCCGAGGTATTCGCACTGGATACGACAGTTCT-3′); and *U6* (Forward: 5′-GGAGACACGCAAACGGAAG-3′, Reverse: 5′-AGTGCAGGGTCCGAGGTATT-3′, RT: 5′-GTCGTATCCAGTGCAGGGTCCGAGGTATTCGCACTGGATACGACTTGGCG-3′). The 2^−ΔΔCt^ method was employed to analyze the mRNA expression levels.

### MTT assay

The transfected MDA-MB-231 and BT549 cells were placed in 96-well plates at a density of 1 × 10^3^ cells/well. Cell viability was assessed at 24, 48, 72, 96, and 120 h of incubation. A 200-μL complete medium was used for cell incubation, with 20 μL MTT (Yeasen, China) added to each well at set time points. After 6 h incubation at 37 °C and 5% CO_2_, the supernatants were removed, and 150 μL DMSO (Sangon Biotech, Shanghai, China) was added to each well to dissolve formazan. The absorbance was quantified at a wavelength of 490 nm.

### Colony formation assay

First, the transfected MDA-MB-231 and BT549 cells were placed in 6-well plates at a density of 5 × 10^2^ cells/well. Second, the cells were incubated for 7–14 days at 37 °C and 5% CO_2_, with the medium changed every 3 days. Third, the medium was removed after colonies formed, and cells were washed thrice with phosphate buffered saline (Servicebio, China) and then fixed with 95% ethanol for 10 min. Finally, the clones were stained with 0.1% crystal violet (Yeasen, China). Representative images were obtained using a camera.

### Transwell assay

The transfected cells (3 × 10^4^) were placed in the upper chamber of Transwell^®^ (Corning Incorporated, Corning, NY, USA) and incubated in serum-free medium, while medium supplemented with 10% fetal bovine serum was added to the lower chamber. After 18 h, the cells that moved to the lower chamber were fixed with 4% paraformaldehyde and stained with 0.05% crystal violet. Representative images were collected using a light microscope (Nikon, Japan).

### Western blot analysis

Proteins were extracted with RIPA lysis buffer (Yeasen, China), and the concentration was measured by BCA Protein Quantification Kit (Yeasen, China). The total protein (30 μg) was resolved by polyacrylamide gel electrophoresis (PAGE), and the separated proteins were transferred to a nitrocellulose membrane (Pall, Port Washington, NY, USA). Following transfer, the membrane was blocked with 5% non-fat milk for 1 h and incubated with the primary antibodies of LATS2 (5888 T, Cell Signalling Technologies [CST], Danvers, MA, USA), Yes-associated protein 1 (YAP1; A1002, ABclonal, Wuhan, China), p-YAP1^S127^(13008 T, CST, USA), CTGF (A11456, ABclonal, China), and β-actin (AC026, ABclonal, China) for 12–18 h at 4 °C. The membrane was washed thrice with Tris Buffered saline-Tween and then incubated with the secondary antibody IRDye^®^ 800CW Goat anti-Rabbit IgG (925-32211, Li-Cor Biosciences, Lincoln, NE, USA) at room temperature for 1 h. The immunoreactive protein bands on the membrane were scanned using Odyssey scanning system (Li-Cor Biosciences, USA).

### Dual-luciferase reporter assay

The dual activity of firefly and Renilla luciferase were detected using Dual-Luciferase Reporter Gene Assay Kit (Promega, Madison, WI, USA), following the manufacturer’s protocol.

### AGO2 immunoprecipitation assay

The AGO2 (10686-1-AP, Proteintech, Chicago, IL, USA) and IgG antibodies (AC005, ABclonal, China) were utilized to observe the interaction between MIR22HG and miR-629-5p using RNA Immunoprecipitation (RIP) Kit (BersinBio, Guangzhou, China).

### Xenograft mouse model

The MDA-MB-231 cells (1 × 10^7^) and transfected cells with stable MIR22HG expression were suspended in 150 μL DMEM (without fetal bovine serum and penicillin/streptomycin) and subcutaneously injected into 6-week-old BALB/c nu/nu female mice. Mice were divided into 2 groups randomly with 4 mice in each group, which were not blinded to investigators. After 30 days, the mice were euthanized, and the tumors were collected. The tumor volume and weight were subsequently measured. All animal experiments were approved by the Animal Care and Use Committee of the Shanghai Tenth People’s Hospital (No. SHDSYY-2020-0600).

### Statistical analysis

The data were presented as means ± standard deviation (SD) and analyzed using GraphPad Prism (version 9.0; GraphPad, San Diego, CA, USA). The variance was similar between the groups that are being statistically compared. Student’s *t* test was used for two-group comparisons, with *P*-values < 0.05 considered as significantly different.

## Data Availability

All data included in this study are available upon request by contact with the corresponding author.
